# Impact of epidermal growth factor and/or β-mercaptoethanol supplementations on the *in vitro* produced buffaloes' embryos

**DOI:** 10.3389/fvets.2023.1138220

**Published:** 2023-03-13

**Authors:** Ahmed Ezzat Ahmed, Ramya Ahmad Sindi, Nasra Ahmed Yousef, Hassan A. Hussein, Magdy R. Badr, Khalid M. Al Syaad, Fatimah A. Al-Saeed, Ahmed Saad A. Hassaneen, Mohamed Abdelrahman, Montaser Elsayed Ali

**Affiliations:** ^1^Department of Biology, College of Science, King Khalid University, Abha, Saudi Arabia; ^2^Department of Theriogenology, Obstetrics, and Artificial Insemination, Faculty of Veterinary Medicine, South Valley University, Qena, Egypt; ^3^Department of Laboratory Medicine, Faculty of Applied Medical Sciences, Umm Al-Qura University, Mecca, Saudi Arabia; ^4^Department of Theriogenology, Faculty of Veterinary Medicine, Assiut University, Assiut, Egypt; ^5^Artificial Insemination and Embryo Transfer Department, Animal Reproduction Research Institute, Al Haram, Giza, Egypt; ^6^Director of the Research Center, Faculty of Science, King Khalid University, Abha, Saudi Arabia; ^7^Department of Biology, College of Science, King Khalid University, Abha, Saudi Arabia; ^8^Research Center for Advanced Materials Science (RCAMS), King Khalid University, Abha, Saudi Arabia; ^9^Key Lab of Agricultural Animal Genetics, Breeding and Reproduction of Ministry of Education, Huazhong Agricultural University, Wuhan, China; ^10^Animal Production Department, Faculty of Agriculture, Assuit University, Assiut, Egypt; ^11^Animal Production Department, Faculty of Agriculture, Al-Azhar University, Assiut, Egypt

**Keywords:** buffalo, IVF, EGF, βME, fertilization, embryo production, oocyte

## Abstract

The present study investigated the effects of epidermal growth factors (EGF) and/or β-Mercaptoethanol (βME) supplementations to oocyte maturation, fertilization, and culture media on the buffalo *in vitro* embryo production. The ovaries were collected and transferred within 2 h to the laboratory. The cumulus oocytes complexes were aspirated from 3 to 8 mm diameter follicles. Firstly, EGF; 0, 10, 20, or 50 ng/mL or βME; 0, 25, 50, 100, or 200 μM were supplemented to the *in vitro* maturation (TCM-199), fertilization (IVF-TALP), or culture (IVC: SOF) media. Our results revealed that supplementing EGF (20 ng/mL) to the TCM-199, IVF-TALP, or SOF media could efficiently improve the growth rates and development of buffalos' embryos, while EGF (50 ng/mL) could stimulate the embryo production only after treatment of the IVF-TALP /or SOF media, but not the IVM medium. However, βME was less efficient than EGF; it stimulated the growth rates of buffalo embryos when supplemented with the maturation and fertilization (IVF-TALP) media in a 50 μM concentration. Secondly, combined EGF (20 ng/mL) and βME (50 μM) were supplemented to the maturation media as effective concentration. The combined treatment of EGF (20 ng/mL) and βME (50 μM) showed no significant enhancing effect on the buffalo embryos compared to each alone. For future perspectives, further study is required to examine the effects of combined EGF and βME on the maturation and fertilization of buffalo oocytes at different categories of age and seasonal localities.

## 1. Introduction

Buffaloes (*Bubalus bubalis*) are important milk and meat producers and efficiently resist adverse environmental conditions. However, buffaloes have lower productivity and reproductive performance than cows due to; anoestrus, delayed puberty, or lower conception rates (1, 2). Compared to cows, the donner buffaloes were known to give lower ovarian follicles numbers, a low embryo recovery rate, and less effective superovulation (3), which may be attributed to the genetic-merits advancements through reproduction-biotechnologies of the cows compared to buffaloes (4, 5). *In-vitro* embryo production (IVEP) is an effective technique for producing high-quality bovine embryos (6).

However, IVEP in buffaloes is limited due to several factors, including low-quality oocytes obtained by ovarian stimulation. Oocyte maturation is essential for fertilization and implantation and completion of meiosis in response to substances naturally originating from follicular fluid and cells (7, 8).

Cavalieri et al. (9), and Marin et al. (10) reported *in vitro* embryo production (IVEP) efficacy was poor as 30–40% of the total oocytes were developed into the blastocyst stage in cattle. However, the blastocyst production rates in buffaloes were usually lower than those achieved in cattle (around 22 vs. 40%, respectively). Thus, different laboratories with scientific and commercial characteristics have worked in the search to improve embryo production rates.

On the other side, Epidermal growth factor (EGF) promotes oocyte maturation, including cellular growth, proliferation, and differentiation and so mitosis stimulation (11) and meiosis induction (12, 13). Also, EGF regulates various ovarian functions, including granulosa and theca cells, follicle-stimulating hormone action, and enhancement of oocyte maturation. Also, Adding EGF alone to IVM media increases the cumulus cell expansion and enhances the nuclear maturation of bovine oocytes. For oocyte maturation and embryo development, antioxidants play a pivotal role in many mammalian species (14) because excessive production of reactive oxygen species negatively affects oocyte maturation and embryo development (15).

β-Mercaptoethanol (βME) is a critical sulphuric amino acid component of oocyte glutathione (GSH) that plays an important role in protection against the toxic effect of oxidative stress. βME is essential to enhance the oocyte GSH synthesis and improve the quality of bovine embryos, and βME can effectively improve oocyte maturation, likely due to the decrease in the oocytes' intracellular free radicals (16). Furthermore, previous studies showed that adding βME solely to the IVM media enhances the expansion of the cumulus cells, the oocyte maturation, and the fertilization rates (17). Also, Sidi et al. (18) reported that βME supplementation could protect the bovine embryos against oxidative stress and enhance the development of embryos with increased blastocysts viability.

EGF and antioxidants could increase the cumulus expansion, helping cellular maturation, but not nuclear (19). Combined supplementation of EGF (20 ng/mL) plus βME (100 μM) in buffalo was proposed to increase the proportion of oocytes with the extruded polar body (20). Similar findings reported significant increases in cleavage and blastocyst rates in response to a combined treatment of EGF and cysteamine, an amino acid component essential for the synthesis of GSH (21). Despite previous studies on supplementing EGF and/or antioxidants to *in-vitro* embryo production media, it is still unclear what is the most effective development stage (IVM, IVF, or IVC) they were involved in buffaloes' embryos. Consequently, the optimum effective concentrations of the EGF and/or βME should be clarified.

The hypothesis of the current study depended on the supplementation of the EGF, βME, or their combination of the IVM (TCM-199), IVF (IVF-TALP), or IVC (SOF) media. The specific aim of this study was to unveil the effects of EGF as a growth promotor and/or βME, as an antioxidant, on the production of buffalo embryos throughout the different developmental stages *in vitro*; maturation, fertilization, and culture.

## 2. Materials and methods

### 2.1. Biological materials

#### 2.1.1. Ovaries

Ovaries were obtained from apparently normal reproductive organs of the slaughterhouse of adult buffaloes (Hide for reviewers). Instantly, the ovaries were placed in a thermos containing warm saline (35°C) with penicillin; 100 IU/mL, and streptomycin; 100 μg/mL, till they reached the laboratory for further processing.

#### 2.1.2. Semen

Frozen 0.25 mL-straws containing 30 × 10^6^ spermatozoa/straw of superior buffalo bulls; obtained from the Animal Reproduction Research Institute (ARRI), El-Haram, Giza, Egypt, were thawed in a water bath (Laboratory Circulating Water Bath HWS-28, China) at 37°C for 30 s. One straw was randomly examined, and at least 50% sperm progressive motility and not exceeding 25% abnormality were approved in the experiment.

#### 2.1.3. Chemicals and media

*In vitro* used media, including; EGF (E-5036), βME (M-6250), and TCM-199 (M-7528), and chemicals used for the preparation of IVF-TALP and SOF media, all were purchased from Sigma-Aldrich (Co. St. Louis County, MO, USA). Other chemicals were obtained from Oxford Laboratory, Maharashtra, India, including; sodium bicarbonate, sodium pyruvate, EDTA, and calcium lactate with reference numbers; S-07990, S-08302, E-03819, and C-02186, respectively. D-glucose (S-2837) was purchased from Alpha Chemika, Mumbai, Maharashtra, India.

### 2.2. Preparation of media

All media were filtered using a 0.2 μm (Millipore, USA) syringe filter and incubated for at least 2 h in a humidified atmosphere (95%) under 5% CO_2_ at 38°C before use.

#### 2.2.1. Aspiration media

Filtered modified phosphate buffer saline (m-PBS, pH 7.1) with 3% (v/v) inactivated fetal calf serum (FCS); tablets soluble in distilled water (six times of distillation) with the addition of Na pyruvate (0.2 mM), glucose (5.56 mM), bovine serum albumin (BSA; 6 mg/mL), streptomycin sulfate (5 mg/100 mL) and penicillin (100 IU/mL) was used as aspiration medium.

#### 2.2.2. Maturation media

Tissue culture media-199 (TCM-199) supplemented with gentamycin (1 μL/mL), Earl's salts, L-glutamine, and 25 mM HEPES with 10% heat-inactivated FCS (PAA, Austria, Linz) were used for oocytes maturation. The media was adjusted for pH 7.3–7.6, osmolarity 270–290 mOsm/kg, and then sterilized by filtration using a 0.22 μm Millipore membrane biological filter.

#### 2.2.3. Sperm capacitation, fertilization, and embryo culture media

Sperm Tyroid's albumin lactate pyruvate medium (sp-TALP) was used for sperm capacitation (22), while IVF-TALP, which contains caffeine as a motility-enhancing substance, was used as fertilization media (23). Modified synthetic oviductal fluid (SOF) medium was prepared at the laboratory according to Tervit et al. (24).

### 2.3. Oocyte recovery

The aspirated oocytes with compact multi-layered cumulus cells and evenly granulated cytoplasm were selected for IVM. The recovered COCs were classified according to their quality into; good oocytes with homogenous granular ooplasm surrounded by more than three-compact layers of cumulus cells, fair oocytes with homogenous ooplasm surrounded by 1–3 layers of cumulus cells, and denuded oocytes with uneven ooplasm (14).

### 2.4. *In vitro* maturation of oocytes

Selected good COCs were washed four times in fresh warm aspiration medium and subjected to the final wash using an IVM medium. In 35-mm Petri dishes, groups of 5–15 COCs were cultured in 50–100 μL droplets of the filtrated IVM media (pH 7.4) covered with sterilized mineral oil for 24 h at 5% CO_2_, 38.5°C, and 90–95% relative humidity (25).

### 2.5. Sperm preparation and capacitation

**Figure 1:**
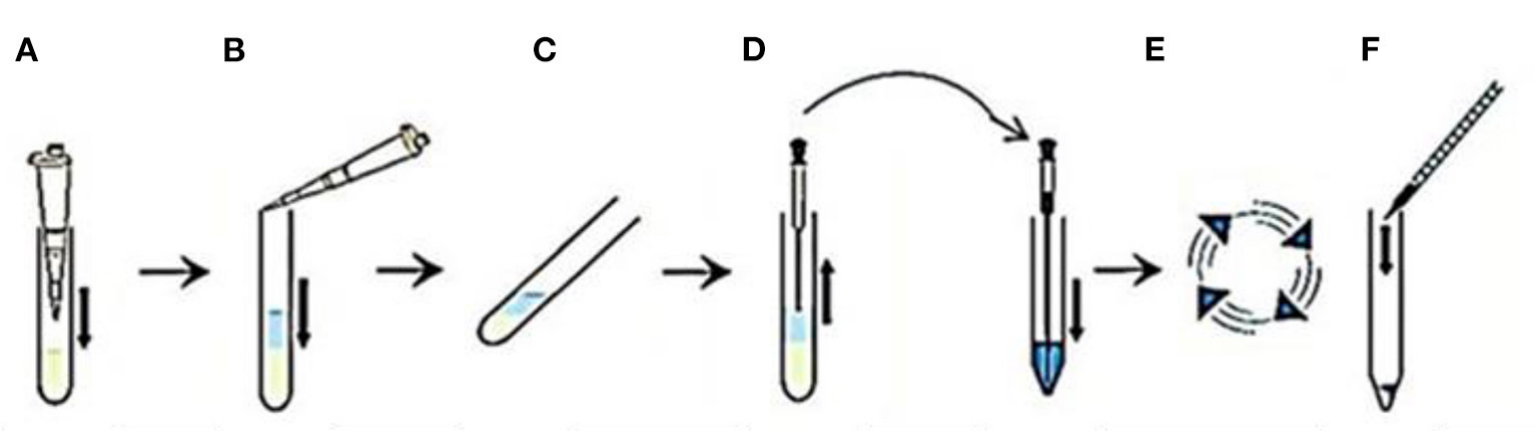
A flow-diagram describing sperm preparation by swim up method using sp-TALP in the following steps. **(A)** One milliliter of semen is added into a centrifuge tube. **(B)** One milliliter of the sp-TALP is added over semen. **(C)** The tube is incubated in an inclined position for 60 min. **(D)** The upper 0.85 mL layer of the motile sperm cells is removed to be placed in a conical tube with adding sp-TALP till reach a final volume of 5 mL. **(E)** Centrifugation for 120 g is performed for 10 min. **(F)** The sperm pellet is resuspended in the culture medium.

Post-thawing, semen aliquots were subjected to a swim-up procedure, as follows, in a 15-ml rounded-bottom centrifuge tube; (a) 1 mL of semen and (b) 1 mL of sp-TALP were added, (c) the tube was incubated at 37°C with 5% CO_2_ for 60 min in a 45° angle, (d) the upper 0.85 mL of supernatant was collected and transferred in a conical tube and diluted with sp-TALP to reach a final volume of 5 mL, (e) the tube was centrifuged at 120 g for 10 min, (f) the supernatant was discarded, and the sperm pellet was diluted with 200 μl of sp-TALP, (g) the recovered spermatozoa were evaluated for progressive motility, and (h) the pellet containing motile population was resuspended in a sperm culture medium and used for culturing with the mature oocytes (26) (Figure 1).

### 2.6. *In vitro* fertilization

**Figure 2:**
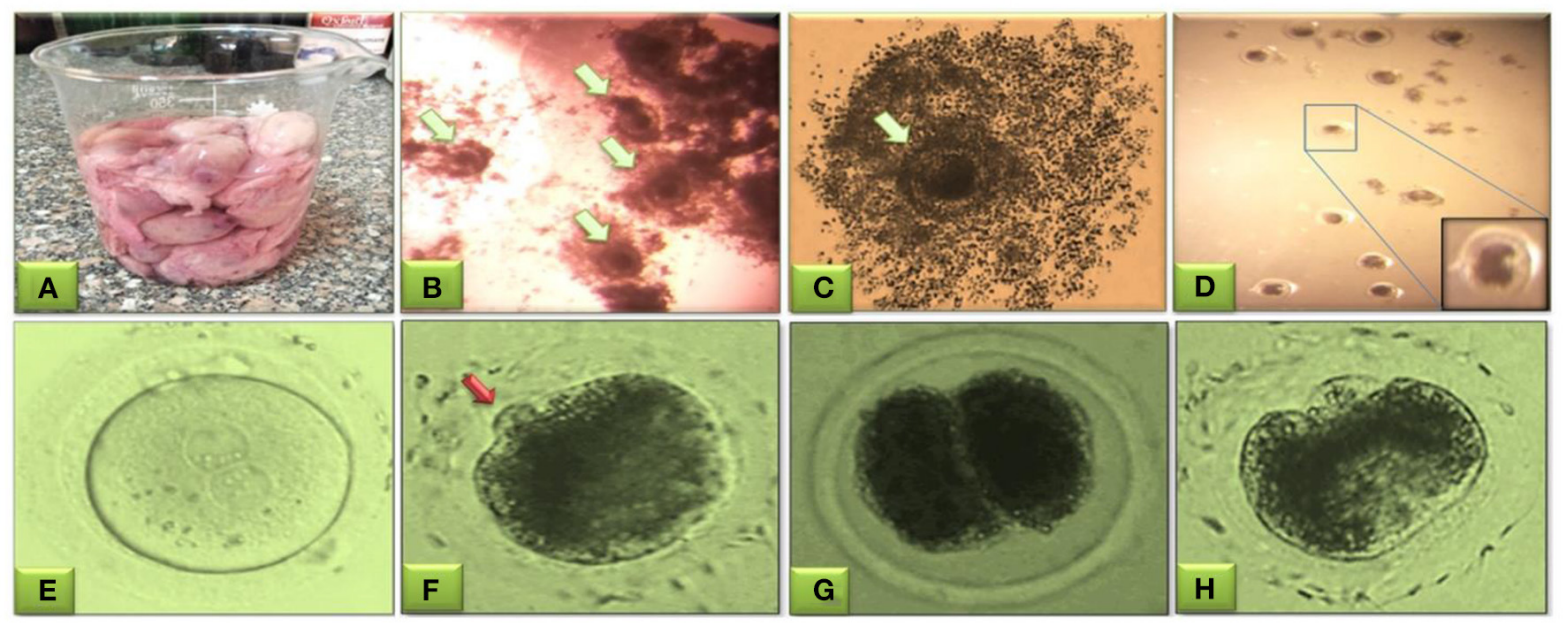
Buffalo oocytes obtained from slaughterhouse ovaries for aspiration of immature cumulus oocytes' complexes (COCs) from follicles of 3–8 mm size **(A)**. Microscopic selection of the good quality even-shaped nucleated oocytes surrounded by expanded cumulus cells (**B, C**, green arrow). Mature oocytes after washing three times in IVF-TALP medium **(D)**. Evaluation of fertilization and cleavage stages in buffalo embryos; fertilized oocyte presenting the male and female pronuclei **(E)**, fertilized oocytes with polar body (red arrow) **(F)**, start of cleavage showing the two-cell stage cleaved embryo **(G)**, and the four-cell stage cleaved embryo **(H)**. This figure is partially retrieved from another recent piolet study (27) based on previous studies (22, 23).

The matured oocytes were partially removed from the surrounding cumulus cells for IVF and then washed twice in a warm IVF-TALP medium. Microdroplet (100 μl) of the IVF-TALP containing caffeine (0.0194 g/mL) and BSA (6 mg/mL) with 10 × 10^6^ sperm/mL was prepared as; 75 μL of IVF-TALP specified to 10 oocytes per 25 μL of sperm suspension. Co-culture of sperm-oocyte for fertilization was performed in a humidified atmosphere (22, 23, 27) (Figure 2).

### 2.7. *In vitro* embryo culture

The presumed zygotes were washed four times in the embryo culture medium, SOF, and cultured in a humidified incubator. Morula production rate was examined for the 8–16 cell stages from 94 to 96 h post-insemination. One day later, 120 h after fertilization, the blastocysts formed and began to appear. A part of SOF-medium was replaced with a fresh one 2–3 times (pre-incubated for at least 2 h) before the embryo transfer to minimize the harmful effect of the waste products elaborated by the embryos (28).

### 2.8. Effects of EGF on oocyte maturation, fertilization, and embryo development

One of four different concentrations of EGF; 0 (control), 10, 20, and 50 ng/mL was supplemented to TCM-199 media during the maturation stage only (first experiment), to IVF-TALP media during the fertilization stage only (second experiment), or to the SOF media during the embryo culturing and development stage only (third experiment).

### 2.9. Effects of βME on oocyte maturation, fertilization, and embryo development

One of the five concentrations of βME; 0 (control), 25, 50, 100, and 200 μM was supplemented to the IVM medium (TCM-199) during the maturation stage only for the first experiment, to the IVF medium (F-TALP) during fertilization only for the second experiment, or to the IVC medium (SOF) during the embryo development stage only for the third experiment.

### 2.10. Effects of combined EGF and βME on the embryo development

After the data of the single use of different concentrations of either EGF or βME as a supplement to one of the three developmental stages, IVM, IVF, or IVC were statistically analyzed, a combined concentration of 20 ng/mL EGF + 50 μM βME was decided as the most effective concentration to be supplemented to the IVM medium (TCM-199) during the maturation stage.

### 2.11. Statistical analysis

Non-parametric data were expressed as percentages of progress, and rates of oocyte maturation and embryo production, morula, and blastocyst were calculated using the Chi-square for trend. Numerical data of the different groups were expressed as mean ± standard error of the mean (SEM) and compared among the three experiments using one-way ANOVA, and Tukey was used as a *post-hoc* test. Each experiment was repeated three times, and the differences were considered for the mean ± SEM for three replicates (*N* = 3). Differences were considered significant at *P* < 0.05. Graph-Pad Prism software was used for analysis (San Diego, USA, V.0.6).

## 3. Results

**Figure 3:**
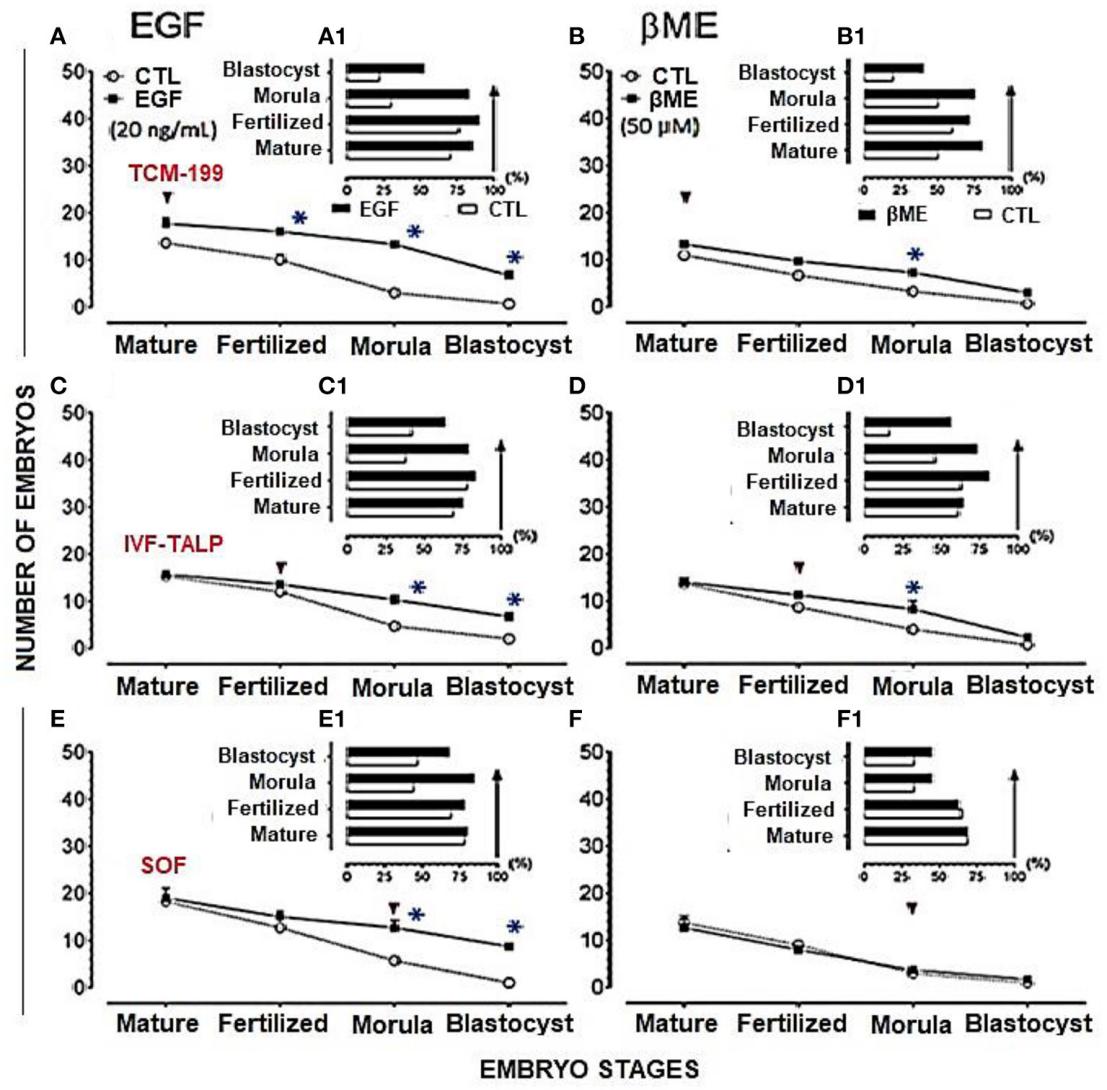
Effects of EGF (20 ng/mL) added to TCM-199, IVF-TALP, and SOF media, on buffalo embryo stages; oocyte maturation, fertilization, morula and blastocyst were shown in **(A, C, E)**, respectively. EGF (20 ng/mL) characteristics on the developmental stages (%) of those embryos *in vitro* were shown in **(A1, C1, E1)**, respectively. Effects of βME (50 μM) added to TCM-199, IVF-TALP, and SOF media, on buffalo embryo stages; oocyte maturation, fertilization, morula and blastocyst were shown in **(B, D, F)**, respectively. βME (50 μM) characteristics on the developmental stages of embryos *in vitro* were shown in **(B1, D1, F1)**, respectively. Values were presented as mean ± SEM for three experiments **(A–F)**. Arrows denotes the stage in treated medium. Asterisk (^*^) denotes significance difference between each respective values (treated vs. CTL) at *P* < 0.05.

Effects of EGF; 10, 20, and 50 ng/mL, supplementation of TCM-199, IVF-TALP, and IVC/SOF media on the development of embryos were shown in Table 1 and Figure 3. The TCM-199, IVF-TALP, or SOF media supplemented with 10 ng/mL of EGF didn't significantly increase the rates of embryo development, morula, and blastocyst compared to each respective control (Table 1). However, supplementation of 20 ng/mL of EGF to the different media significantly increased the stages of embryonic development up to the blastocyst stage compared to each respective control, as shown in the demographic trends of Chi^2^ analyses (Table 1 and Figures 3A1, C1, E1, respectively; *P* < 0.05). Embryo production rates were also significantly increased in response to supplementing the IVF-TALP and SOF media compared to their controls' trend (Chi^2^ = 4.4 and 7.5, respectively; *P* < 0.05), but not the TCM-199 media, with 50 ng/mL of EGF (Table 1).

**Table 1:** Effects of epidermal growth factor (EGF) added to maturation (TCM-199), fertilization, (IVF-TALP), and culture (SOF) media on the embryo stages in buffaloes; oocyte maturation, fertilization, cleavage/morula and blastocyst development.

**EGF concentrations**	**Media treatment with EGF**											
**10 ng/mL**	**TCM-199**				**IVF-TALP**				**SOF**			
	**CTL**		**10 ng/mL**		**CTL**		**10 ng/mL**		**CTL**		**10 ng/mL**	
	***N***	**%**	***N***	**%**	***N***	**%**	***N***	**%**	***N***	**%**	***N***	**%**
Total original oocytes	55		48		66		62		70		67	
Mature oocytes	39	70.9	37	77.1	46	69.7	48	77.4	55	78.6	52	77.6
Fertilized oocytes	30	76.9	29	78.4	36	78.3	38	79.2	38	69.1	39	75.0
Morula production	9	30.0	18	62.1	14	38.9	20	52.6	17	44.7	22	56.4
Blastocyst production	2	22.2	6	33.3	6	42.9	11	55.0	8	47.1	11	50.0
Total number (100%)	103				128				137			
Chi^2^ (*P*-value)	3.8 (0.053)^NS^				2.0 (0.157)^NS^				0.95 (0.329)^NS^			
**20 ng/mL**	**CTL**		**20 ng/mL**		**CTL**		**20 ng/mL**		**CTL**		**20 ng/mL**	
	***N***	**%**	***N***	**%**	***N***	**%**	***N***	**%**	***N***	**%**	***N***	**%**
Total original oocytes	55		62		66		62		70		71	
Mature oocytes	39	70.9	53	85.5	46	69.7	47	75.8	55	78.6	57	80.3
Fertilized oocytes	30	76.9	48	90.6	36	78.3	39	83.1	38	69.1	45	78.9
Morula production	9	30.0	40	83.3	14	38.9	31	79.5	17	44.7	38	84.4
Blastocyst production	2	22.2	21	52.5	6	42.9	20	64.5	8	47.1	26	68.4
Total number (100%)	117				128				141			
Chi^2^ (*P*-value)	19.0 (< 0.0001)^***^				9.3 (0.002)^**^				10.0 (0.001)^**^			
**50 ng/mL**	**CTL**		**50 ng/mL**		**CTL**		**50 ng/mL**		**CTL**		**50 ng/mL**	
	***N***	**%**	***N***	**%**	***N***	**%**	***N***	**%**	***N***	**%**	***N***	**%**
Total original oocytes	55		54		66		57		70		67	
Mature oocytes	39	70.9	38	70.4	46	69.7	46	80.7	55	78.6	54	80.6
Fertilized oocytes	30	76.9	28	73.7	36	78.3	35	76.1	38	69.1	41	75.9
Morula production	9	30.0	13	21.4	14	38.9	25	71.4	17	44.7	35	85.4
Blastocyst production	2	22.2	4	66.7	6	42.9	12	48.0	8	47.1	20	57.1
Total number (100%)	109				123				137			
Chi^2^ (*P*-value)	0.54 (0.462)^NS^				4.4 (0.037)^*^				7.5 (0.006)^**^			

The differences were considered significant at ^*^P < 0.05, ^**^P < 0.01, ^***^P < 0.001; ^NS^ stands for non-significant difference. CTL, control. EGF was added to the three media in 10, 20, or 50 ng/mL. The values denotes sum of three experiments. Chi-square (Chi^2^) for the trend of the morula and blastocyst production rates was calculated considering the sum of values scored for the three experiments.

Fertilized oocytes proportion with the extruded 2nd polar body was the highest in TCM-199 supplemented with 20 ng/mL of EGF (90.6%) compared to control (76.9%) (16.0 ± 6.0 vs. 10.0 ± 1.2; *P* < 0.05). Furthermore, morula production was significantly increased in response to the same concentration of EGF added to the TCM-199 media (83.3%) rather than the control (30.0%; 13.3 ± 0.7 vs. 3.0 ± 0.6; Table 1 and Figures 3A, A1; *P* < 0.05).

The effects of βME supplementation 25, 50, 100, and 200 μM to the TCM-199, F-TALP, or SOF media on the buffalo embryo development are shown in Table 2 and Figure 3. Interestingly, adding 50 μM βME to the maturation or fertilization media significantly increased the development trend of morula and blastocyst stages compared to their controls (Chi^2^ = 12.0, *P* = 0.0005, and Chi^2^ = 9.5, *P* = 0.002, respectively; Table 2 and Figures 3B1, D1, respectively). The other concentrations of βME, 25, 100, and 200 μM, didn't increase the rates of oocyte maturation, fertilization, or cleavages of embryos compared to those of the control (Table 2). According to Chi^2^ analyses, 50 μM of βME significantly scored the highest trend for morula production after supplementation of the IVM (75.9 vs. 50.0%) or IVF medium (73.5 vs. 46.2%) compared to their controls (Table 2 and Figures 3B1, D1; *P* < 0.001). Morula production was significantly increased in response to the same concentration of βME added to the TCM-199 media (75.9%) rather the control (50.0%; 7.3 ± 0.3 vs. 3.3 ± 0.3; Table 2 and Figures 3B, D; *P* < 0.05).

**Table 2:** Effects of Beta-Mercaptoethanol (βME) added to TCM-199, IVF-TALP, and SOF media on the embryo stages in buffaloes; oocyte maturation, fertilization, cleavage/morula, and blastocyst development.

**βME concentrations**	**Media treated with** β**ME**											
**25** μ**M**	**TCM-199**				**IVF-TALP**				**SOF**			
	**CTL**		**25** μ**M**		**CTL**		**25** μ**M**		**CTL**		**25** μ**M**	
	***N***	**%**	***N***	**%**	***N***	**%**	***N***	**%**	***N***	**%**	***N***	**%**
Total original oocytes	65		58		67		63		59		60	
Mature oocytes	33	50.8	36	62.1	41	61.2	40	63.5	41	69.5	44	73.3
Fertilized oocytes	20	60.6	23	63.9	26	63.4	31	77.5	27	65.9	31	70.5
Morula production	10	50.0	15	65.2	12	46.2	20	64.5	9	33.3	12	38.7
Blastocyst production	2	20.0	5	33.3	2	16.7	6	30.0	3	33.3	3	25.0
Total number (100%)	123				130				119			
Chi^2^ (*P*-value)	2.6 (0.107)^NS^				3.4 (0.064)^NS^				0.26 (0.61)^NS^			
**50** μ**M**	**CTL**		**50** μ**M**		**CTL**		**50** μ**M**		**CTL**		**50** μ**M**	
	***N***	**%**	***N***	**%**	***N***	**%**	***N***	**%**	***N***	**%**	***N***	**%**
Total original oocytes	65		49		67		65		59		55	
Mature oocytes	33	50.8	40	81.6	41	61.2	42	64.6	41	69.5	38	69.1
Fertilized oocytes	20	60.6	29	72.5	26	63.4	34	81.1	27	65.9	24	63.2
Morula production	10	50.0	22	75.9	12	46.2	25	73.5	9	33.3	11	45.8
Blastocyst production	2	20.0	9	40.9	2	16.7	14	56.0	3	33.3	5	45.6
Total number (100%)	114				132				114			
Chi^2^ (*P*-value)	12.0 (0.0005)^***^				9.5 (0.002)^**^				0.38 (0.536)^NS^			
**100** μ**M**	**CTL**		**100** μ**M**		**CTL**		**100** μ**M**		**CTL**		**100** μ**M**	
	***N***	**%**	***N***	**%**	***N***	**%**	***N***	**%**	***N***	**%**	***N***	**%**
Total original oocytes	65		67		67		60		59		61	
Mature oocytes	33	50.8	42	62.7	41	61.2	42	70.0	41	69.5	42	68.1
Fertilized oocytes	20	60.6	25	59.5	26	63.4	33	78.6	27	65.9	27	64.3
Morula production	10	50.0	16	64.0	12	46.2	12	36.4	9	33.3	11	40.7
Blastocyst production	2	20.0	6	37.5	2	16.7	3	25.0	3	33.3	4	36.4
Total number (100%)	132				127				120			
Chi^2^ (*P*-value)	2.2 (0.13)^NS^				0.89 (0.35)^NS^				0.08 (0.774)^NS^			
**200** μ**M**	**CTL**		**200** μ**M**		**CTL**		**200** μ**M**		**CTL**		**200** μ**M**	
	***N***	**%**	***N***	**%**	***N***	**%**	***N***	**%**	***N***	**%**	***N***	**%**
Total original oocytes	65		59		67		75		59		63	
Mature oocytes	33	50.8	33	55.9	41	61.2	42	56.0	41	69.5	45	71.4
Fertilized oocytes	20	60.6	15	45.5	26	63.4	28	66.7	27	65.9	29	64.4
Morula production	10	50.0	8	53.3	12	46.2	7	25.0	9	33.3	10	34.5
Blastocyst production	2	20.0	2	25.0	2	16.7	2	28.6	3	33.3	1	10.0
Total number (100 %)	124				142				122			
Chi^2^ (*P*-value)	0.10 (0.75)^NS^				0.77 (0.38)^NS^				0.10 (0.746)^NS^			

The differences were considered significant at ^**^P ≤ 0.01; ^***^P ≤ 0.001. ^NS^Stands for non-significant difference. βME was added to the three media in 25, 50, 100, or 200 μM. The values denotes sum of three experiments. Chi^2^ for trend of embryo production was calculated considering the sum of values scored for the three experiments.

**Figure 4:**
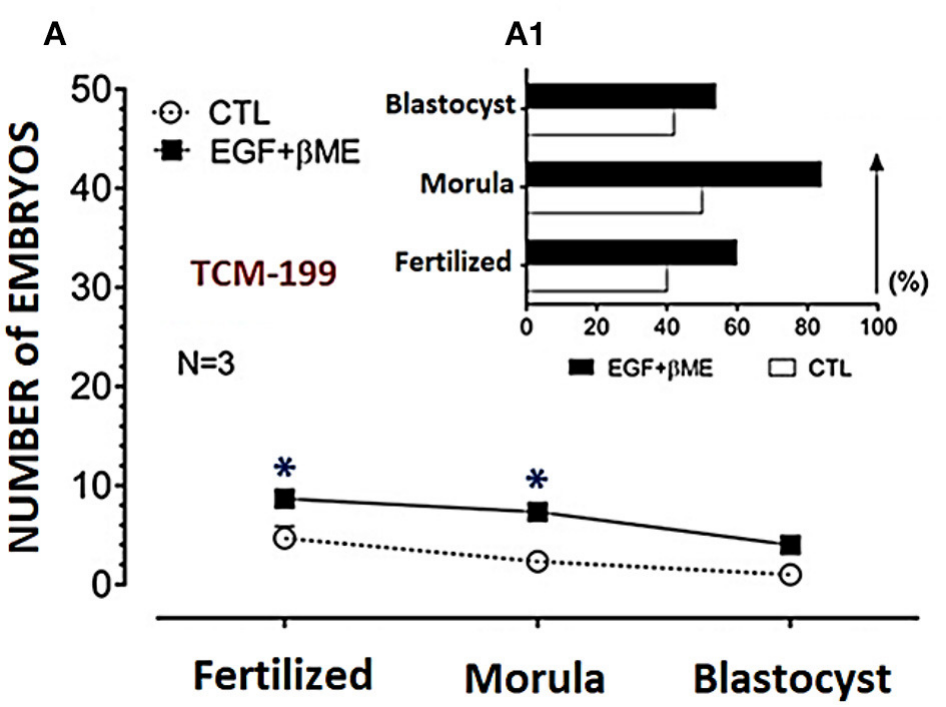
Effects of combined supplementation of EGF (20 ng/mL) and βME (50 μM) to the TCM-199 medium on the buffalo embryo stages; fertilized oocytes, morula, and blastocyst, were shown in **(A)**, and the trend demograph for the blastocyst production was shown in **(A1)**. Asterisk (^*^) denotes significance difference between each respective values (treated vs. CTL) at *P* < 0.05.

Effective concentrations of either the EGF or βME showed higher potency in the TCM-199 media compared to the fertilization or culture media. Therefore, the effects of combined EGF (20 ng/mL) plus βME (50 μM) supplementation of IVM/TCM-199 media on embryo development were evaluated (Table 3 and Figure 4). Supplementation of the TCM-199 media with a combined concentration of EGF and βME significantly increased the fertilization and growth rates of morula and blastocyst compared to the control (Table 3 and Figures 4A, A1; Chi^2^ = 6.0, *P* = 0.015).

**Table 3:** Effects of combined EGF and βME supplementations to the TCM-199 medium on the embryo stages; cleavage and gastrulation.

**Embryo stages**	**TCM-199 medium**			
	**CTL**		**EGF** + β**ME**	
	***N***	**%**	***N***	**%**
Total original oocytes	35		43	
Fertilized oocytes	14	40.0	26	60.5
Morula production	7	50.0	22	84.6
Blastocyst production	3	42.9	12	54.5
Total number (100%)	78			
Chi^2^ (*P*-value)	6.0 (0.015)^*^			

EGF (20 ng/mL) and βME (50 μM) were added to the three media. Chi^2^ for trend of embryo production was calculated. The differences were considered significant at ^*^(*P* < 0.05). Other explanations were given in Tables 1, 2. The values denotes sum of three experiments.

The demographic characteristics of *in vitro* maturation, fertilization, and cleavage rates in addition to blastocyst production in response to EGF and/or βME added to the different media using Chi^2^ analyses have shown that EGF in 20 ng/mL was the highly potent agent rather than βME 50 μM. Although the combined treatment of both EGF and βME significantly increased the growth rates of those embryos, it didn't score additional enhancement compared to those of EGF or βME, each alone (Chi^2^ = 19.0 and 12.0, respectively; Tables 1, 2 and Figures 3, 4, respectively; *P* < 0.05). Accordingly, considering the blastocyst to morula ratio, the proportion of blastocyst production scored the highest trend in the TCM-199 medium supplemented with EGF (20 ng/mL) rather, in order, than those supplemented with βME (50 μM) and then the combined supplementation; EGF (20 ng/mL) plus βME (50 μM), compared to those of controls (52.5 vs. 22.2, 40.9 vs. 20.0, and 54.5 vs. 42.9%, respectively). However, considering the ratio of the blastocyst to the original oocytes, the proportion of blastocyst production also scored the highest trend after the treatment of TCM-199 medium with EGF rather, in order, than the combined supplementation of EGF plus βME, and then βME compared with each respective control (33.9 vs. 3.6, 27.9 vs. 8.6, and 18.4 vs. 3.1%, respectively; Tables 1–3 and Figures 3, 4, respectively; *P* < 0.05).

## 4. Discussion

The present study investigated the effects of supplementing EGF- and/or βME to the TCM-199, IVF-TALP, or SOF media on the *in vitro* growth rates of the buffalo embryos. The study demonstrated that the optimum concentration of EGF as 20 ng/mL supplemented to TCM-199, IVF-TALP, or SOF media, was potent for enhancing the expansion of COCs, inducing higher maturation, fertilization, cleavage, morula, and blastocyst production (19). However, Sadeesh et al. (20) reported that the optimum concentration of EGF was also 20 ng/mL added to the maturation/TCM-199 medium for buffalo's *in-vitro-*produced embryos. Earlier studies reported that oocyte competence and blastocyst development rate were improved at 5 ng/mL of EGF (29). Moreover, Other *in-vitro* embryo production studies in bovine found that the oocytes' maturation rates were increased at 10 ng/mL of EGF added to the maturation media (30). Previous studies were compatible with our findings that higher concentrations of EGF, 40 or 50 ng/mL, reduced the blastocyst development rather than the 20 ng/mL, which may be attributed to a suggested phenomenon of growth factor-induced receptor down-regulation. Generally, EGF was previously reported to enhance the cellular function causing early embryo development in mammals; more specifically, our study elucidated that the optimum way to add EGF only as a supplement to *in-vitro*-produced buffaloes' embryos was 20 ng/mL added to the TCM-199 media (14).

Our findings successfully determined that the optimum concentration of βME alone was 50 μM added to the TCM-199 media according to those reported by Songsasen and Apimeteetumrong (31), who showed that supplementation of TCM-199 medium with βME increased the proportion of buffaloes' oocytes exhibiting synchronous pronuclei formation and enhance the development of the produced blastocysts. Our results agreed with those of Furnus et al. (32), who provided evidence that the optimal development of cattle embryos *in vitro* partially depends on the presence of precursor amino acids for intracellular GSH production during IVM. Similarly, the present study provides the beneficial effect of βME as likely attributed to the function of βME in the production of GSH to improve the buffalo embryo maturation and fertilization by increasing the blastocyst production rates.

A previous study in porcine showed that supplementation of lower concentration of βME, 25 μM, in the IVM medium increased the oocyte maturation rate (33); however, 50 μM of cysteamine, or βME, could enhance not only the oocyte maturation but also the transformation of sperm pronuclei formation (34). Our findings revealed that supplementing 100 or 200 μM of βME to the maturation medium couldn't improve the growth of buffaloes' oocytes. Those findings agreed with Patel et al. (35), who reported similar findings *in-vitro*-produced buffaloes. Cleavage, morula, and blastocyst rates were increased in the media containing 50 μM of βME rather than those of control which agreed with Sadeesh et al. (20), who stated that supplementation of antioxidants such as βME to the culture medium enhanced the antioxidant systems within the embryo and stimulated the embryonic development. Thus, the oocyte developmental competence could be improved by increasing the antioxidant capacity of the oocytes during maturation *in vitro* (36, 37). The present results are supported by Sidi et al. (18) studies which reported that low molecular weight thiol-compounds like the cysteamine or βME could stimulate the synthesis of oocyte GSH. Both cysteamine and βME reduce cystine into cysteine and promote cysteine uptake to enhance the oocyte's GSH synthesis. This pathway explains the efficacy of βME in improving oocyte maturation (16).

Although βME added to SOF media showed no effect on the embryo's development, Sidi et al. (18) showed that supplementation of βME into the culture media could protect the bovine embryos against oxidative stress and promote both amino acid transport, DNA synthesis, and development of embryos with increased the blastocysts viability. A previous report by Kobayashi et al. (38) found that adding βME in 100 μM to the TCM-199 improved the blastocyst formation and the cell numbers of porcine oocytes, which may likely be due to the species difference.

The present study reported that a combination of 20 ng/mL of EGF plus 50 μM of βME to the TCM-199 media significantly improved the maturation of buffalo oocytes *in vitro*. The combination of EGF and antioxidants could increase the maturation rate due to cumulus expansion, not nuclear maturation. A significant increase in cleavage and blastocyst rates in response to a combined treatment of EGF and cysteamine was reported (21). On the other hand, Singhal et al. (19) reported that the TCM-199 medium supplemented with EGF (20 ng/mL) plus cysteamine (50 μM) showed no effect on the buffalo oocyte maturation and embryo development. Still, in agreement with our study, the oocyte cleavage rate was improved (46.4%) after supplementation of the maturation medium with 20 ng/mL of EGF plus 50 μM of βME.

Sadeesh et al. (20) stated that combined supplementation of EGF; 20 ng/mL plus βME; 100 μM, in the maturation medium improved the maturation of buffalo oocytes with polar bodies and further embryo development *in vitro*. They also compared the TALP medium with the Bracket and Oliphant (BO) medium for culturing and blastocyst production. Their findings were consistent with our study, which used the combined treatments, but the βME was at 50 μM since all stages of the embryo development and cleavage rates were improved compared to their respective controls. Another study testing the use of BO medium instead of IVF-TALP should be carried out in the future. They also observed that increasing the dose caused a decrement in the cumulus expansion and the polar body formation rates.

Simultaneously, in our study, using EGF or βME each alone rather than combined use could be more effective for cleavage and blastocyst production. These results could be attributed to the embryos' receptor's down-regulation, supporting what Sadeesh et al. (20) reported, but the potency was different than ours.

Growth factors activate the receptor tyrosine kinases (RTKs) on the cell's surface, which triggers cellular proliferation by stimulating the tyrosine autophosphorylation of RTKs and recruiting the Src homology-2 (SH2) domains. Those activated RTKs are rapidly internalized into cells by endocytosis and eventually degraded by the lysosomes in a process termed receptor down-regulation, which plays a crucial role in terminating the cellular proliferation signals and preventing cellular overgrowth (39). The epidermal growth factor receptor (EGFR) is the RTK (40). Activation of the protein kinase C, which phosphorylates EGF-receptor threonine (41), or stimulation of the calmodulin-dependent protein kinase II, which phosphorylates serine in those receptors (42) leads to regulation of the EGFRs.

A recent study showed that EGF and EGFR are expressed in the bovine oocytes and their zona pellucida, in addition to the follicular granulosa, cumulus cells, follicular fluid, and theca folliculi (43). As far as we know, studies concerning the cellular effects and their RTKs in response to βME maybe not be available. However, our finding suggests a pivotal role of βME in the up/down-regulation of the EGFR, which explain the decreased growth rates of the buffalo embryos after supplementing the EGF with βME. Also, the regulatory mechanism of RTKs could be variable between the embryonic stages; oocyte, morula, and blastocyst, which explains the variant ratios detected between blastocysts vs. morulae or blastocyst vs. oocytes. The results of Sadeesh et al. (20) and our study are consistent but different in potency. We approve that the higher doses lead to adverse effects. They observed the adverse effect on the maturation, but in ours, it happened in the culturing and blastocyst production between the single and combined treatments.

## 5. Conclusions

In conclusion, EGF could efficiently improve the growth rates and development of the *in vitro* produced buffalo embryos after supplementation of the TCM-199, IVF-TALP, or SOF media with the potent concentration of 20 ng/mL, while the increased concentrations of EGF; up to 50 ng/mL, stimulate the embryo production only after treatment of the fertilization/ and/ or culture media, but not that the maturation media. However, βME was less efficient than EGF; it could only stimulate the embryos' growth rates after treatment of the TCM-199 and IVF-TALP media with a concentration of 50 μM. Furthermore, the combined treatment of both EGF and βME showed no enhancement effect on the buffalo embryo production compared to each alone. Our study strongly supports the phenomenon of growth factor-induced receptor downregulation.

## Data availability statement

The raw data supporting the conclusions of this article will be made available by the authors, without undue reservation.

## Ethics statement

This study was discussed and approved by the Ethical Research Committee members at the Faculty of Veterinary Medicine, South Valley University, Qena, Egypt (No. 57/18.09.2022).

## Author contributions

AA and NY: conceptualization, methodology, data curation, and writing original draft. RS, HH, MB, KS, FA-S, and AH: methodology, writing-review, and editing, and critical reading. MA and MEA: editing, critical reading, data curation, formal analysis. All authors listed in this paper have contributed to the preparation and execution of this research. All authors have read and agreed to the published version of the manuscript.

## References

[1] Nandi S Raghu HM Ravindranatha BM Chauhan MS. Production of buffalo (*Bubalus bubalis*) embryos *in vitro*: premises and promises. Reprod Domest Anim. (2002) 37:65–74. 10.1046/j.1439-0531.2002.00340.x 11975742

[2] El Sabry MI Almasri O. Space allowance: a tool for improving behavior, milk and meat production, and reproduction performance of buffalo in different housing systems-a review. Trop Anim Health Prod. (2022) 54:266. 10.1007/s11250-022-03247-y 35970907 PMC9378332

[3] Yuan X Shi W Jiang J Li Z Fu P Yang C. Comparative metabolomics analysis of milk components between Italian Mediterranean buffaloes and Chinese Holstein cows based on LC-MS/MS technology. PLoS ONE. (2022) 17:e0262878. 10.1371/journal.pone.0262878 35077464 PMC8789157

[4] Mapletoft RJ Bennett Steward K Adams GP. Recent advances in the superovulation in cattle. Reprod Nutr Dev. (2002) 42:601–11. 10.1051/rnd:2002046 12625424

[5] Chen AJ Loya F. Strengthening goal-directed functioning after traumatic brain injury. Handb Clin Neurol. (2019) 163:435–56. 10.1016/B978-0-12-8042816.00023-9 31590745

[6] Currin L Baldassarre H de Macedo MP Glanzner WG Gutierrez K Lazaris K. Factors affecting the efficiency of *in vitro* embryo production in prepubertal mediterranean water buffalo. Animals. (2022) 12:3549. 10.3390/ani12243549 36552466 PMC9774791

[7] Sutton-McDowall ML Gilchrist RB Thompson JG. Effect of hexoses and gonadotropin supplementation on bovine oocyte nuclear maturation during *in vitro* maturation in a synthetic follicle fluid medium. Reprod Fertil Dev. (2005) 17:407–15. 10.1071/RD04135 15899152

[8] Seneda MM Zangirolamo AF Bergamo LZ Morotti F. Follicular wave synchronization prior to ovum pick-up. Theriogenology. (2020) 150:180–5. 10.1016/j.theriogenology.2020.01.024 31982155

[9] Cavalieri FLB Morotti F Seneda MM Colombo AHB Andreazzi MA Emanuelli IP. Improvement of bovine *in vitro* embryo production by ovarian follicular wave synchronisation prior to ovum pick-up. Theriogenology. (2018) 117:57–60. 10.1016/j.theriogenology.2017.11.026 29198975

[10] Marin DFD De Souza EB De Brito VC Nascimento CV Ramos AS Filho STR. *In vitro* embryo production in buffaloes: from the laboratory to the farm. Anim Reprod. (2019) 16:260–6. 10.21451/1984-3143-AR2018-0135 33224285 PMC7673586

[11] Richani D Gilchrist RB. The epidermal growth factor network: role in oocyte growth, maturation and developmental competence. Hum Reprod Update. (2018) 24:1–14. 10.1093/humupd/dmx029 29029246

[12] Bolamba D Russ KD Harper SA Sandler JL Durrant BS. Effects of epidermal growth factor and hormones on granulosa expansion and nuclear maturation of dog oocytes *in vitro*. Theriogenology. (2006) 65:1037–47. 10.1016/j.theriogenology.2005.06.017 16169071

[13] Lindbloom S Farmerie T Clay C Seidel G Carnevale E. Potential involvement of EGF-like growth factors and phosphodiesterases in initiation of equine oocyte maturation. Anim Reprod Sci. (2008) 103:187–92. 10.1016/j.anireprosci.2007.04.006 17507186

[14] Yang XY Jia ZW. [The role of EGF-like factor signaling pathway in granulosa cells in regulation of oocyte maturation and development]. Yi Chuan. (2019) 41:137–45. 10.16288/j.yczz.18-193 30803944

[15] Takahashi M. Oxidative stress and redox regulation *in vitro* development of mammalian embryos. J Reprod Dev. (2012) 58:1–9. 10.1262/jrd.11-138N 22450278

[16] Moussa M Yang CY Zheng HY Li MQ Yu NQ Yan SF. Vitrification alters cell adhesion related genes in pre-implantation buffalo embryos: protective role of β-mercaptoethanol. Theriogenology. (2019) 125:317–23. 10.1016/j.theriogenology.2018.11.013 30502624

[17] Shang JH Huang YJ Zhang XF Huang FX Qin J. Effect of ß-mercaptoethanol and buffalo follicular fluid on fertilisation and subsequent embryonic development of water buffalo (*Bubalus bubalis*) oocytes derived from *in vitro* maturation. Ital J Anim Sci;. (2007) 6:751–4. 10.4081/ijas.2007.s2.751

[18] Sidi S Pascottini OB Angel-Velez D Azari-Dolatabad N Pavani KC Residiwati G. Lycopene supplementation to serum-free maturation medium improves *in vitro* bovine embryo development and quality and modulates embryonic transcriptomic profile. Antioxidants (Basel). (2022) 11:344. 10.3390/antiox11020344 35204226 PMC8868338

[19] Singhal S Prasad S Prasad JK Gupta HP. Effect of including growth factors and antioxidants in maturation medium used for *in vitro* culture of buffalo oocytes recovered *in vivo*. Anim Reprod Sci. (2009) 113:44–50. 10.1016/j.anireprosci.2008.05.078 18620823

[20] Max MC Bizarro-Silva C Búfalo I González SM Lindquist AG Gomes RG. In vitro culture supplementation of EGF for improving the survival of equine preantral follicles. In Vitro Cell Dev Biol Anim. (2018) 54:687–91. 10.1007/s11626-018-0296-9 30284096

[21] El-Ratel IT Fouda SF. Potentiality of epidermal growth factor or/and cysteamine in maturation medium on *in vitro* rabbit embryo production and apoptosis. Global Vet. (2016) 17:505–12. 10.5829/idosi.gv.2016.505.512

[22] Parrish JJ Susko-Parrish JL Leibfried-Rutledge ML Critser ES Eyestone WH First NL. Bovine i*n vitro* fertilisation with frozen-thawed semen. Theriogenology. (1986) 25:591–600. 10.1016/0093-691X(86)90143-3 16726150

[23] Galli C Lazzari G. Embryo technologies in dairy cattle. In: The 26th Eur Holst Red Holst Conference Proceedings. (2015). p. 1–20.

[24] Tervit HR Whittingham DG Rowson LE. Successful culture *in-vitro* of sheep and cattle ova. J Reprod Fertil. (1972) 30:493–7. 10.1530/jrf.0.0300493 4672493

[25] Kadoom AK Abdel-Khalek AE Shamiah Sh M El-Sharawy ME Abd El-Razek IM. In vitro maturation, fertilisation and development of prepubertal and mature buffalo oocytes. Egypt J Anim Prod. (2014) 51:65–9. 10.21608/ejap.2014.93652

[26] Allamaneni SS Agarwal A Rama S Ranganathan P Sharma RK. Comparative study on density gradients and swim-up preparation techniques utilizing neat and cryopreserved spermatozoa. Asian J Androl. (2005) 7:86–92. 10.1111/j.1745-7262.2005.00008.x 15685358

[27] Yousef NA Hussein HA Badr MR Ezzat A. Effect of epidermal growth factors (EGF) on the maturation and developmental competence of buffalo's oocytes and embryo stages *in vitro*. SVU-IJVS. (2018) 1:85–94. 10.21608/svu.2018.19870

[28] Aoyagi Y Fukui Y Iwazumi Y Urakawa M Tgono H. Effect of culture systems on development of *in vitro* fertilised bovine ova to blastocysts. Theriogenology. (1990) 34:749–59. 10.1016/0093-691X(90)90029-S 16726878

[29] Sirisathien S Hernandez-Fonseca HJ Brackett BG. Influences of epidermal growth factor and insulin-like growth factor-I on bovine blastocyst development *in vitro*. Anim Reprod Sci. (2003) 77:21–32. 10.1016/S0378-4320(02)00272-5 12654525

[30] Mtango NR Varisanga MD Dong YJ Rajamahendran R Suzuki T. Growth factors and growth hormone enhance *in vitro* embryo production and post–thaw survival of vitrified bovine blastocyst. Theriogenology. (2003) 59:1393–402. 10.1016/S0093-691X(02)01163-9 12527085

[31] Songsasen N Apimeteetumrong M. Effects of beta-mercaptoethanol on formation of pronuclei and developmental competence of swamp buffalo oocytes. Anim Reprod Sci. (2002) 71:193–202. 10.1016/S0378-4320(02)00064-7 12047928

[32] Furnus CC de Matos DG Picco S García PP Inda AM Mattioli G. Metabolic requirements associated with GSH synthesis during *in vitro* maturation of cattle oocytes. Anim Reprod Sci. (2008) 109:88–99. 10.1016/j.anireprosci.2007.12.003 18242890

[33] Choe C Yong-Won S Eun-Jin K Cho SR Hyun-Jong K Sun-Ho C. Synergistic effects of glutathione and Beta-mercaptoethanol treatment during *in vitro* maturation of porcine oocytes on early embryonic development in a culture system supplemented with L-cysteine. J Reprod Dev. (2010) 56:575–82. 10.1262/jrd.09-214H 20657156

[34] Gonçalves FS Barretto LS Arruda RP Perri SH Mingoti GZ. Effect of antioxidants during bovine *in vitro* fertilisation procedures on spermatozoa and embryo development. Reprod Domest Anim. (2010) 45:129–35. 10.1111/j.1439-0531.2008.01272.x 18992086

[35] Patel PA Chaudhary SS Puri G Singh VK Odedara AB. Effects of β-mercaptoethanol on *in vitro* maturation and glutathione level of buffalo oocytes. Vet World. (2015) 8:213–6. 10.14202/vetworld.2015.213-216 27047075 PMC4774706

[36] Ali ME Hassan AT Daghash AH Fahmy S. Follicular diameters and progesterone level in Egyptian ewe lambs using flushing and some hormonal treatments. Arch Agric Sci J. (2019) 2:22–30. 10.21608/aasj.2019.16416.1009

[37] Hussein HA Hassaneen ASA Ali ME Sindi RA Ashour AM Fahmy SM. The impact of rumen-protected l-arginine oral supplementation on libido, semen quality, reproductive organ biometry, and serum biochemical parameters of rams. Front Vet Sci. (2022) 9:899434. 10.3389/fvets.2022.899434 35812886 PMC9263849

[38] Kobayashi M Lee ES Fukui Y. Cysteamine or β-mercaptoethanol added to a defined maturation medium improves blastocyst formation of porcine oocytes after intracytoplasmic sperm injection. Theriogenol. (2006) 65:1191–9. 10.1016/j.theriogenology.2005.06.019 16154628

[39] Mizuno E Iura T Mukai A Yoshimori T Kitamura N Komada M. Regulation of epidermal growth factor receptor down-regulation by UBPY-mediated deubiquitination at endosomes. Mol Biol Cell. (2005) 16:5163–74. 10.1091/mbc.e05-06-0560 16120644 PMC1266416

[40] Wee P Wang Z. Epidermal growth factor receptor cell proliferation signaling pathways. Cancers. (2017) 9:52. 10.3390/cancers9050052 28513565 PMC5447962

[41] Davis RJ. Independent mechanisms account for the regulation by protein kinase C of the epidermal growth factor receptor affinity and tyrosine-protein kinase activity. J Biol Chem. (1988) 263:9462–9. 10.1016/S0021-9258(19)76563-6 3379075

[42] Countaway JL. Nairng AC, Davis RJ. Mechanism of desensitisation of the epidermal growth factor receptor protein-tyrosine kinase. J Biol Chem. (1992) 267:1129–40. 10.1016/S0021-9258(18)48406-2 1309762

[43] Luo Y Zhang R Gao J Wang Y Zhang W Qing S. The localisation and expression of epidermal growth factor and epidermal growth factor receptor in bovine ovary during oestrous cycle. Reprod Dom Anim. (2020) 55:822–32. 10.1111/rda.13690 32330337

